# Principal causes of acute poisoning in an emergency service: experience between 2014 and 2021 at a University Hospital in Southwestern Colombia

**DOI:** 10.1038/s41598-024-54159-w

**Published:** 2024-02-12

**Authors:** David Hurtado, Jaime A. Quintero, Yeraldin Alejandra Rodríguez, Daniel Esteban Pérez, Roger Figueroa Paz, Julio Diez-Sepúlveda

**Affiliations:** 1 Departamento de Medicina de Emergencias, Fundación Valle del Lili, Carrera 98 No.18-49, 760032 Cali, Colombia; 2 Facultad de Ciencias de la Salud, Universidad Icesi, Calle 18 # 122-135, Cali, Colombia; 3 Centro de Investigaciones Clínicas (CIC), Fundación Valle del Lili, Carrera 98 No.18-49, 760032 Cali, Colombia; 4 Facultad de Ciencias de la Salud, Semillero de Investigación en Medicina de Emergencias y Reanimación (SIMER), Calle 18 # 122-135, Cali, Colombia

**Keywords:** Acute, Poisoning, Emergency room, Toxic, Epidemiology, Public health, Physical examination

## Abstract

Acute poisonings are a global public health problem, which implies costs and disease burden for society. In Colombia, there is a significant underreporting of data on acute poisoning and data gaps on the toxicological profile of the population. This study aims to identify the epidemiology of acute poisoning in a high-complexity hospital in southwestern Colombia. A descriptive study with retrospective data collection was performed. The variables were expressed through the measure of central tendency and dispersion. Categorical variables were described in proportions. A total of 406 patients were included. The median age was 31 years (IQR 23–48), 56.2% were male, and only 19.2% had a history of mental illness. Suicidal intent represented 58.8% of the cases, and the most frequent route of exposure was the oral route (81.6%). The most prevalent groups of substances were pesticides (34.2%) and medicines (32%). The most common etiological agent was organophosphates (16.5%). Cholinergic toxidrome was the most common. The average stay in the ICU was 4.5 days (± 4.8), and the mortality was 4.2%. The principal causes of acute poisoning were drugs and pesticides, with a predominant etiology of organophosphates and depressants of the central nervous system. There was a significant predominance of young male patients with suicidal intent, low mental disorders, elevated unemployment rate, and similar mortality reported in other studies. This study improves the knowledge about acute poisoning in southwestern Colombian to carry out multicenter analytic studies.

## Introduction

Acute poisoning is a frequent cause of consultation in emergency services, impacting public health. According to the World Health Organization (WHO), poisonings caused around 106,683 deaths in 2016^1^. The burden of disease attributed to environmental exposure and handling of chemical substances amounts to 4.9 million deaths (83% of the global burden of diseases) and 86 million disability-adjusted life years^2^. Acute poisonings imply a high cost for health systems and society. One study reported that care for poisoning and its implications after hospitalization required approximately 812.5 million dollars for people between 25 and 64 years of age^3^.

In Colombia, 33.165 cases of acute poisoning occurred in 2020. The principal causes were medications, psychoactive substances, and pesticides^4^. However, there is significant underreporting without reliable epidemiological data on the toxicology profile and clinical outcomes. Therefore, strict monitoring of acute poisoning is justified, given the impact on morbidity and mortality and the high cost. This study aimed to describe the epidemiology of patients admitted with acute poisoning to the emergency department of a high-complexity hospital in southwestern Colombia between 2014 and 2021.

## Material and methods

This retrospective cross-sectional descriptive study was derived from the institutional poisoning registry of the Fundación Valle del Lili University Hospital (LILITOX). This registry began in May 2022 with an average annual admission of 33 patients. Our hospital is a center of national reference for the management of poisoning patients in southwestern Colombian. Our study included all patients with acute poisoning seen in the emergency room between 2014 and 2021.

### Population

All patients older than 17 years of age with the following diagnoses were included (T36–T50 Poisoning by drugs, T51 Toxic effect of alcohol, T52 Toxic effect of organic solvents, T53 Toxic effect of halogenated derivatives of aliphatic and aromatic hydrocarbons, T54 Effect Toxic effect of corrosive substances, T55 Toxic effect of detergents and soaps, T56 Toxic effect of metals, T57 Toxic effect of other inorganic substances, T58 Toxic effect of carbon monoxide, T59 Toxic effect of other gases, fumes, and vapors, T60 Toxic effect of Pesticides [Pesticides], T61 Toxic effect of harmful substances ingested as seafood, T62 Toxic effect of other harmful substances ingested as food, T63 Toxic effect of contact with poisonous animals, T64 Toxic effect of aflatoxin and other mycotoxins contaminating food, T65 Toxic effect of other and unspecified substances).

Patients under palliative care were excluded due to their heightened susceptibility to adverse outcomes, complications, and mortality, introducing potential bias to the results. Pregnant patients were excluded from the study due to their initial point of care was the obstetric unit. Moreover, this population group is classified as vulnerable and was consequently omitted from consideration. Patients with chronic exposure of poisoning were not included due to the non-urgent nature of their management and follow-up.

We included sociodemographic variables such as sex, age, comorbidities and occupation, clinical variables, the time between exposure, general management and use of antidotes, group of substances involved, decontamination, type of exposure, route of exposure, referral site, and particular agent involved. The clinical outcomes were status at the end of care (alive, dead, referred), days of hospitalization and ICU stay, the requirement for invasive, non-invasive mechanical ventilation or both, vasoactive support, and hemodialysis.

The classification of the hospitals is according to Resolution number 5261 of 1994 of the Ministry of Health and Social Protection of Colombia. This resolution classifies care centers into four levels of complexity to define the responsibility of health personnel in each of them^16^.

First-level hospital has a general doctor, auxiliary personnel, paramedics, or other non-specialized health professionals. These hospitals provided minor surgical procedures and laboratory services available for general analysis.

The second-level hospital has a general doctor and medical specialists (internal medicine, obstetrics, pediatrics, general surgery, anesthesia and orthopedics). These hospitals provided surgical procedures of low and medium complexity.

Third and fourth-level hospitals have highly specialized staff and technical equipment—for example, cardiology, intensive care unit, and specialized imaging units. These hospitals provided surgical procedures of medium and high complexity, including transplants at Level IV.

Self-medication was related with misusing a drug inconsistent with dosing recommendations or/and for an incorrect indication.

Hemodynamic instability was defined as the patient presenting hypotension (SBP < 90 mmHg, DBP < 60 mmHg, MAP < 65 mmHg), a weak pulse, distal coldness, prolonged capillary refill, difficulty breathing, altered state of consciousness, decreased urinary output, tachycardia, and increased lactate.

Timely administration of antidotes was defined as the time lapse in minutes from exposure to agent to administration of antidote.

### Statistical analysis

The normality of the variables was determined through a Shapiro–Wilk test. The quantitative variables were expressed through measures of central tendency and dispersion. Categorical variables were described in proportions.

### Ethics approval and consent to participate

The Institutional Review Board at the Hospital (Full name of the ethics committee: Comité de Ética en Investigación Biomédica de la Fundación Valle del Lili) approved this study (Registration No. 210-2018, approved on August 1, 2018. Letter No. 161-2021). This study adheres to the Helsinki Declaration of Ethical Principles for Medical Research Involving Human Subjects. According to resolution 8430 of 1993 of the Colombian Ministry of Health, this study did not represent risk and not required an informed consent. This study adhered to the standards of the STROBE guideline. The researchers did not expose the patients to biological, psychological, or social risks. Therefore, the ethics committee approved the waiver of informed consent.

## Results

**Figure 1:**
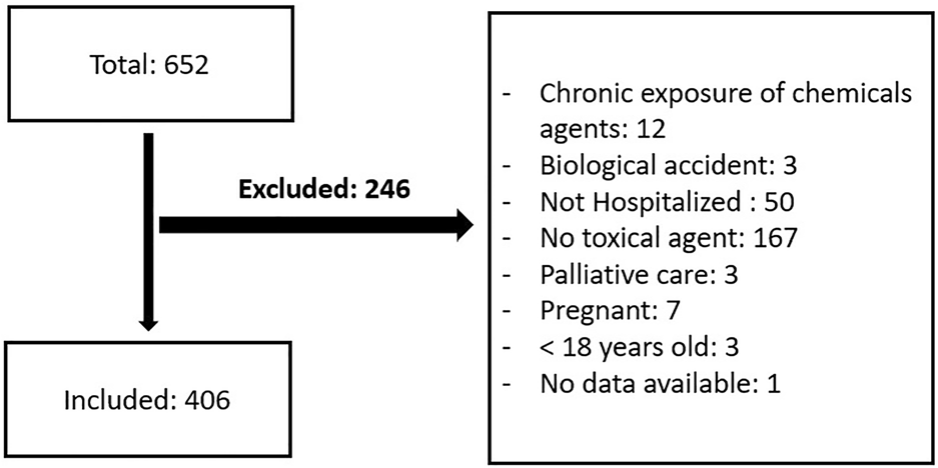
Flowchart.

A total of 406 patients were included (Fig. 1). Men represented 56.2% of patients. The median age was 31 years (IQR 23–48). The most frequent place of occurrence was at home in 70.19%. Eighty-one percent of cases came from the urban area. The oral route was the most common route of intoxication (81%) (Table 1).

**Table 1:** Sociodemographic and clinical characteristics.

Total of patients	Total, n = 406	Urban, n: 329	Rural, n:77
Age* (years)	31 (23–48)	32 (24–48)	28 (21—50)
Gender			
Male	228 (56.2)	166 (50.5)	62 (80.5)
Female	178 (43.8)	163 (49.5)	15 (19.5)
Medical history			
Mental disorders	79 (19.5)	72 (21.9)	7 (9.1)
Cardiovascular diseases	51 (12.6)	45 (13.7)	6 (7.8)
Neurological disorders	25 (6.2)	21 (6.4)	4 (5.2)
Psychoactive drugs	20 (4.9)	17 (5.2)	3 (3.9)
Hypothyroidism	15 (3.7)	14 (4.3)	1 (1.3)
Diabetes	12 (3.0)	11 (3.3)	1 (1.3)
Gastrointestinal diseases	11 (2.7)	10 (3.0)	1 (1.3)
Renal diseases	9 (2.2)	9 (2.7)	0
Cancer	8 (2.0)	8 (2.4)	0
Inmuno-rheumatological disorders	7 (1.7)	3 (0.9)	1 (1.3)
Respiratory diseases	7 (1.7)	6 (1.8)	1 (1.3)
Dyslipidemia	4 (1.0)	4 (1.2)	0
Immunosuppression	3 (0.7)	6 (1.8)	0
Previous poisoning	51 (12.6)	43 (13.1)	8 (10.4)
Region of origin/occurrence			
Valle del Cauca	318 (78.3)	299 (90.9)	19 (24.7)
Cauca	84 (20.7)	27 (8.2)	57 (74.0)
Others	4 (1.2)	3 (0.9)	1 (1.3)
Level referral of hospital			
First-level hospital	343 (84.5)	288 (87.5)	55 (71.4)
Second-level hospital	42 (10.3)	21 (6.4)	21 (27.3)
Third-level hospital	6 (1.5)	5 (1.5)	1 (1.3)
Not apply	15 (3.7)	15 (4.6)	0
Employment status			
Unknown	116 (28.6)	103 (31.3)	13 (16.9)
Informal job	125 (30.8)	76 (23.1)	49 (63.6)
Without work	89 (21.9)	78 (23.7)	11 (14.3)
Professional job	76 (18.7)	72 (21.9)	4 (5.2)
Place of occurrence			
Home	285 (70.2)	233 (70.8)	52
Workplace	46 (11.3)	38 (11.6)	8 (10.4)
Street	31 (7.6)	26 (7.9)	5 (6.5)
Mall	15 (3.7)	15 (4.6)	0
Unknown	12 (3.0)	10 (3.0)	2 (2.6)
Rural area	11 (2.7)	1 (0.3)	10 (13)
Educational places	5 (1.2)	5 (1.5)	0
Military installations	1 (0.3)	1 (0.3)	0
Route of exposure			
Oral	319 (81.6)	259 (78.7)	60 (77.9)
Respiratory	59 (11.2)	43 (13.1)	16 (20.8)
Skin	10 (2.6)	10 (3.0)	0
Parenteral	10 (2.6)	10 (3.0)	0
Unknown	8 (2.0)	7 (2.1)	1 (1.3)
Clinical variables			
Cardiac frequency**	93.09 (± 24.60)	92.45 (± 23.9)	95.81 (± 27.43)
Respiratory frequency**	19.68 (± 5.93)	19.09 (± 5.41)	22.2 (± 7.29)
Systolic blood pressure**	121.72 (± 28.09)	121.04 (± 26.19)	124.61 (± 35.15)
Diastolic blood pressure**	72.84 (± 18.84)	73.30 (± 17.96)	70.9 (± 22.25)
Oxygen saturation			
> 92%	343 (84.5)	288 (87.5)	55 (71.4)
85–92%	29 (7.1)	24 (7.4)	5 (6.5)
70–85%	20 (4.9)	8 (2.4)	12 (15.6)
< 70%	14 (3.4)	9 (2.7)	5 (6.5)
Temperature (°C)			
35 a 37	397 (97.8)	323 (98.2)	74 (96.1)
38 a 40	5 (1.2)	4 (1.2)	1 (1.3)
< 34	4 (1.0)	2 (0.6)	2 (2.6)
Altered mental status	207 (51.0)	159 (48.3)	48 (62.3)
CNS excitation^#^	15 ( 7.2)	11 (3.3)	3 (3.9)
CNS depression^#^	192 (92.8)	148 (45.0)	45 (58.4)
Electrocardiogram	117 (28.8)	93 (28.3)	24 (31.2)
Normal^#^	68 (58.1)	57 (61.3)	11 (45.8)
Abnormal^#^	49 (41.9)	36 (38.7)	13 (54.2)

*Median, IQR.

**Mean, standard deviation.

^#^Percentages calculated excluding patients for whom we did not have data for the variable in question.

Mental illness was the most common medical history followed by cardiovascular and neurological diseases. Patients accepted from first-level hospitals represented 84.5%.

The group of substances involved was mainly pesticides (34.2%), followed by medicines (32%) and chemical substances (8.9%). In 117 patients, an electrocardiogram was obtained. Only 41.9% presented some alteration without specifying what type of findings (Table 2).

**Table 2:** Group of substances and attention times.

	Total, n = 406	Urban, n: 329	Rural, n: 77
Pesticides	139 (34.2)	84 (25.5)	55 (71.4)
Medications	130 (32.0)	127 (38.6)	3 (3.9)
Chemical substances	36 (8.9)	35 (10.6)	1 (1.3)
Psychoactive drugs	29 (7.1)	29 (8.8)	0
Alcohols	22 (5.4)	18 (5.5)	4 (5.2)
Venoms	15 (3.7)	2 (0.6)	13 (16.9)
Toxic gases	14 (3.4)	14 (4.3)	0
Solvents	11 (2.7)	9 (2.7)	2 (2.6)
Food	9 (2.2)	9 (2.7)	0
Heavy metals	4 (1.0)	4 (1.2)	0
Time between toxical agent exposure and consultation (min)** (n: 203)^#^	120 (60–300)	120 (60–263)	203 (125–308)
Time between toxical agent exposure and first aid poisoning management (min)** (n: 195)^#^	180 (84–348)	166 (80–336)	249.5 (174–383)
Time between toxical agent exposure and antidote administration (min)** (n: 61)^#^	287 (133–610)	186.5 (121.5–425.5)	420 (287–774)

**Mean, standard deviation.

^#^Percentages calculated excluding patients for whom we did not have data for the variable in question.

**Figure 2:**
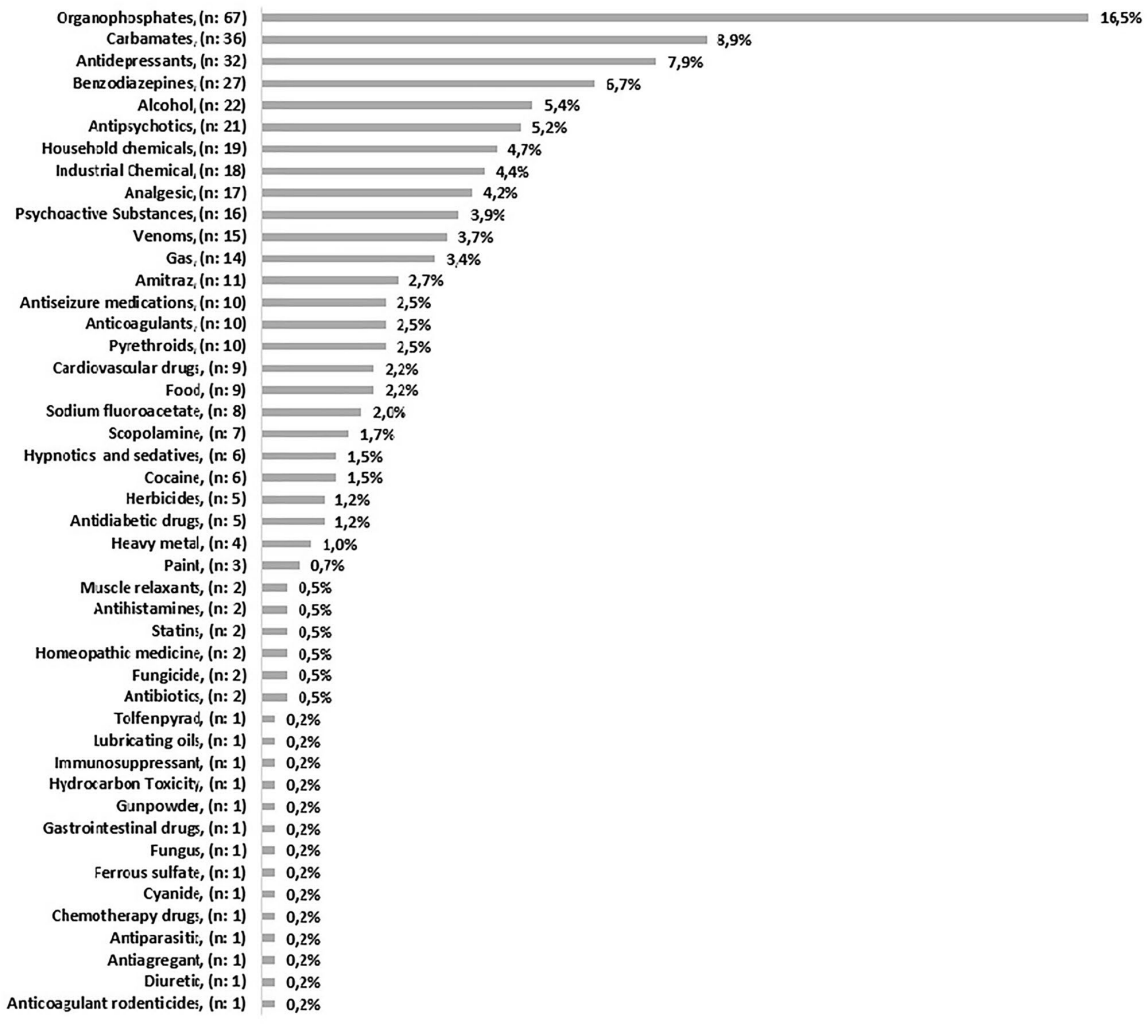
Toxics agents.

The main etiological agents were organophosphates, followed by carbamates, antidepressants, and benzodiazepines (Fig. 2). Of the pesticide groups, organophosphates and carbamates were the pesticides most related to poisoning, and the toxical agent most common was organophosphate (47.06%), followed by pesticides (35.29%).

The times between exposure to the toxical agent and the first consultation, immediate clinical management, and the administration of an antidote are described in Table 2. Regarding first aid for poisoning, 160 patients received decontamination measures, 366 concomitant standard medical management and 158 required an antidote.

**Figure 3:**
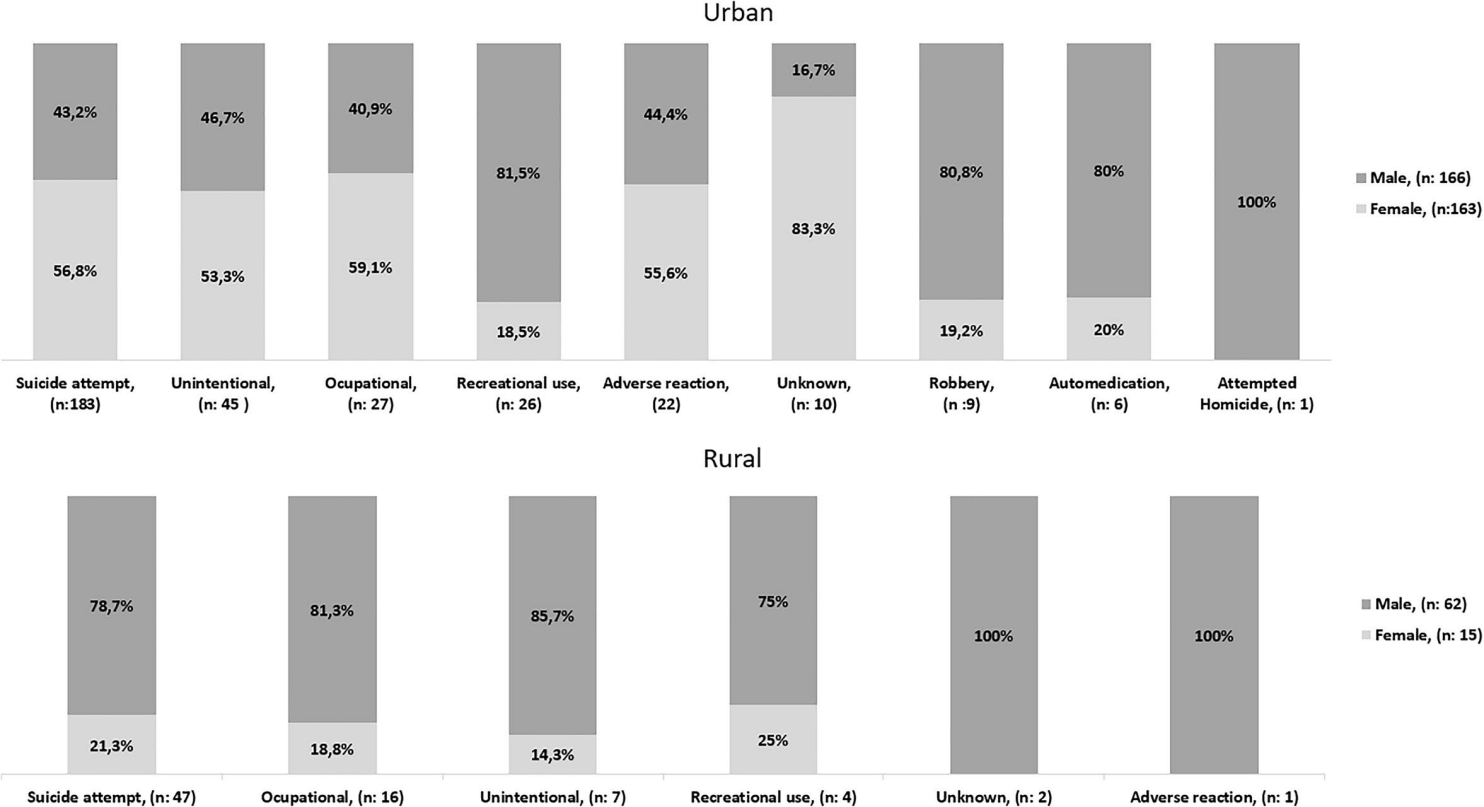
Types of exposure in rural and urban environments.

Suicidal intent and occupational exposure occurred most frequently in areas of rural origin, especially in men. In urban areas, the most frequent exposure types were suicidal intent and unintentional, especially in women. The exposure types unknown, robbery and attempted Homicide were not present in the rural population (Fig. 3).

**Figure 4:**
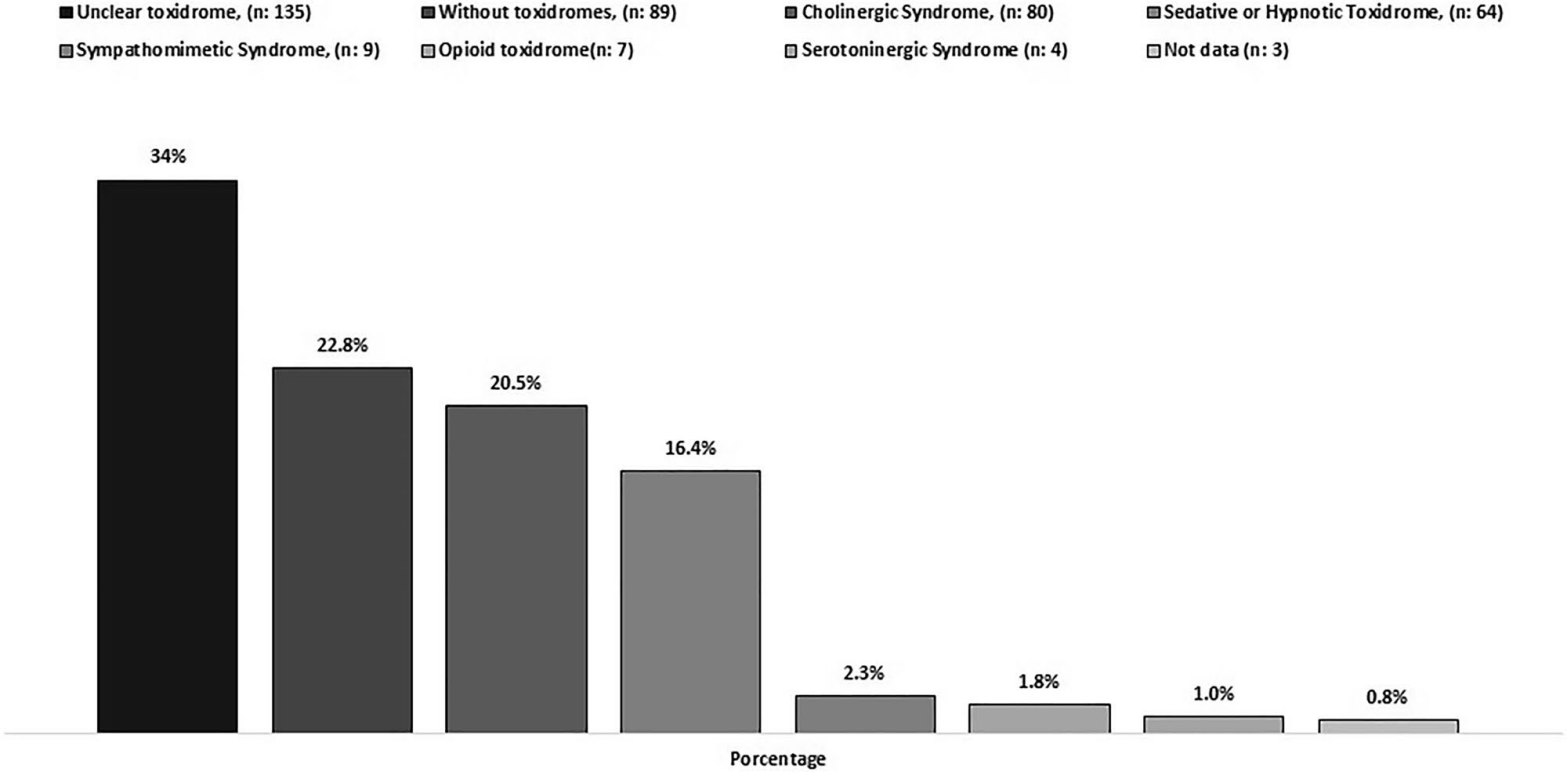
Toxidromes syndromes.

A toxidrome syndrome could not be determined in 34% of the cases. Cholinergic syndrome occurred in 20.5% of cases (Fig. 4).

One hundred patients required invasive mechanical ventilation with a mean time of 3.8 days (± 4.86). Only 24 patients required non-invasive mechanical ventilation with a mean duration of 2.04 days (± 1.96). About 9.1% of the patients admitted with macro-hemodynamic alteration due to hypotension or tachycardia required vasoactive support (Table 3).

**Table 3:** Outcomes.

Management clinical	Total, n = 406	Urban, n: 329	Rural, n: 77
Decontamination measures, (gastric lavage and activated charcoal were the decontamination measures provided)	160 (39.9)^#^	120 (37.0)^#^	40 (51.9)
Standard medical management	367 (90.4)	293 (89.1)	74 (96.1)
Invasive mechanical ventilation	100 (24.6)	61 (18.5)	39 (50.6)
Duration of invasive mechanical ventilation days**	3.8 (± 4.86)	3.5 (± 3.85)	4.2 (± 6.16)
Noninvasive mechanical ventilation	24 (5.9)	17 (5.2)	6 (7.8)
Duration of noninvasive mechanical ventilation**	2.04 (± 1.96)	1.9 (± 1.85)	2.3 (± 2.42)
Vasoactive	37 (9.1)	23 (7.0)	14 (18.2)
Hemodialysis	16 (3.9)	11 (3.3)	5 (6.4)
Hemodynamic instability	135 (33.2)	102 (31)	33 (42.9)
Hospitalization area after 24 h of arrival			
Observation unite	191 (47.0)	172 (52.3)	19 (24.7)
Intensive care unit	186 (45.8)	129 (39.2)	57 (74.0)
General medicine ward	29 (7.1)	28 (8.5)	1 (1.3)
Average length of stay in the intensive care unit (days)**	4.4 (± 4.8)	4.1 (± 4.6)	5.2 (± 5.2)
Total time of intra hospital care (days)**	4.5 (± 6.5)	4.1 (± 6.2)	6.4 (± 7.5)
Referral to another hospital	64 (15.8)	47 (14.7)	17 (22.1)
Death	17 (4.2)	10 (3.0)	7 (9.1)
Toxic agent related to death			
Organophosphates	4 (23.5)	0	4 (57.1)
Analgesic	2 (11.8)	1 (10)	1 (14.3)
Anticoagulants	2 (11.8)	2 (20)	0
Venoms	2 (11.8)	2 (20)	0
Alcohol	1 (5.9)	1 (10)	0
Antidepressants	1 (5.9)	0	1 (14.3)
Antipsychotics	1 (5.9)	0	1 (14.3)
Carbamates	1 (5.9)	1 (10)	0
Gas	1 (5.9)	1 (10)	0
Herbicides	1 (5.9)	1 (10)	0
Psychoactive substances	1 (5.9)	1 (10)	0
Cause related to death			
Occupational	2 (11.8)	0	2 (28.6)
Unintentional	3 (17.6)	2 (20)	1 (14.3)
Suicide attempt	9 (52.9)	6 (60)	3 (42.9)
Adverse reaction	1 (5.9)	0	1 (14.3)
Recreational use	2 (11.8)	2 (20)	0

**Mean, standard deviation.

^#^Percentages calculated excluding patients for whom we did not have data for the variable in question.

In the first 24 h, most patients were treated in the emergency room (47%), followed by the intensive care unit (45.8%). The average length of stay in the intensive care unit was 4.4 days (± 4.8), and the total hospital management time was 4.5 days (± 6.5). Mortality was 4.2%. In this group, the most frequent specific toxic agent related to mortality were organophosphates (23.5%) follow by carbamates and herbicides. The mortality associated with suicidal intent was 52.9% (Table 3).

## Discussion

Our study aimed to identify the main causes of acute poisoning attended in the emergency department between 2014 and 2021. Toxic agents involved in acute poisoning were medicines, pesticides, and chemical substances. The agents responsible for the poisoning were pesticides (organophosphates and carbamates), medications that depress the central nervous system (benzodiazepines), and antidepressants. Less than 50% of the patients required ICU management in the first 24 h. The mortality was less than 5% of the population related to the use of medications and pesticides.

Naseri et al. performed a cross-sectional study that included patients admitted to the Imam Reza Hospital in Birjand between 2017 and 2018 to address this knowledge gap by examining the admissions in a major poisoning center in eastern Iran^5^. More than half were women, and the main cause of poisoning was accidental. Medications were the most common poisoning agent, followed by narcotics, and chemicals. The urban population represented 56.5%. The results of this study are very similar to our study in relation to the greater number of women in urban areas. However, the main difference was the related cause of poisoning, which was suicide, and that we did not include the minor population. They found that in the subgroup of suicide attempters (minors), education was a variable to consider.

In our study the frequency of these events concerning the mental illness group and the use of psychoactive substances as triggering causes of the events are low. Perhaps, this finding is related to the type of employment because not having it or having informal jobs represented more than 50% of the cases and only 18% had a professional job. This contrast with the poverty index in our region described a high level of informal employment (67.5%), low education (39.4%), school lag (23.8%), long-term unemployment (12.1%), and health insurance (10.2%) during 2018^6^. Another factor to explain this outcome is the underreporting of mental illness diagnoses due to the social stigma, and it could contribute to suicidal ideation^7^.

Recently, Rageh et al. performed a study to provide the profile and outcomes of common poisons in the mid-region of the Nile Delta^8^. A total of 9.713 patients were included. Female gender was the most common (55.9%), and the suicidal intent topped the causes of poisoning (58.6%) followed by accidental poisoning (34.7%). Rodenticides, drug overdoses, and chemical substances were the causative agents with the highest incidence. Intentional poisoning was caused by rodenticides (39.3%), Central nervous system-abused pharmaceutical drugs (22.3%), and other pharmaceutical drugs (20.4%). Accidental poisoning was usually associated with chemicals (26.5%) and CNS-abused pharmaceutical drugs (20.7%). Rodenticides, chemicals, insecticides, and animal envenomation were significantly more frequent among males. In the USA, abuse (51.9%) has been described as the principal cause of poisoning, followed by suicidal intent in 23.6%^9^. Compared with this study, our results reveal a higher proportion of men, and the most common type of exposure was suicidal intent in both genders. Although the Rageh sample was significantly superior, our study demonstrates a similar distribution of the main cause of poisoning. However, our study describes a higher proportion of men in rural areas but with an elevated number of women in urban areas. These results can be sustained with the latest bulletin on mental health and suicidal behavior in Colombia in 2018 which describes that being a man and living in a rural area increases the probability of suicide^10^.

Pastó et al. evaluated the epidemiologic changes of acute intoxications between 1994 and 2004. They described a similar proportion between men and women with a mean age of 32 years. Drugs (benzodiazepines) were the most common poisoning agent followed by alcohol. The principal cause of poisoning was suicidal intent increased in the last years^11^. However, the medications were the second most common poisoning agent, and psychoactive drugs were the fourth cause. These differences concerning the toxic agents can be associated with the type of employment related to the field and the free sale of pesticides, which should be evaluated by local authorities.

The second agent responsible for the poisoning was medications. A study in New Zealand described that the medicines most involved in medication poisoning were paracetamol, antidepressants, and quetiapine^12^. In the US, the annual report of the national poisoning data system informed that during 2021 the substances mainly involved were analgesics (11.2%), household cleaning substances (10.7%), cosmetic products (5.88%), antidepressants (5.61%) and antipsychotics (4.73%), predominating the group of medications^9^. In our country, the national statistics reported that pesticides, medicines, and psychoactive substances were the principal causes of acute poisoning in 2020^10^. Likewise, a descriptive study showed that the principal causes were psychoactive substances (41.1%), medications (27.5%), and pesticides (24.8%)^13^. Our study documented that antidepressants and benzodiazepines were the medications involved in poisoning. This result is due to least 19% of our population having a history of mental illness.

Usually, some patients arrive at the hospital late to receive the initial clinical management, which is crucial to avoid complications and adverse outcomes. Kaya et al. described the clinical of acute poisoning in 2010 in Duzce City. The mean time for admission to the emergency service for patients with food intoxication after the incident was 142 min, for those with drug intoxication 173 min, and for those with undefined intoxication 289 min^14^. In our study, the median care time for patients with acute poisoning from exposure to the start of first aid for poisoning was 180 min (84–348). Our results showed that 95% of the patients admitted were referred from first and second-level hospitals, which could explain the variability in the care time.

The first and second-level hospitals realized first aid management, including decontamination (Gastric lavage and Activated charcoal) measures in 39.4% (160 patients). In the United States, two studies described that the use of activated charcoal was 1.97% and 3.6%, gastric lavage 0.08% and 0.04%, and total intestinal irrigation 0.08 and 0.11%^9,15^. Lund C et al. described that 9–16% used decontamination measures in their study^16^. Studies in Russia and India described that the use of this measure was 22% and 35%^17,18^. In Bolivia, an organophosphates study evidenced that gastric lavage in patients with intoxication was 96%^19^. Currently, the available evidence about decontamination measures does not impact morbidity and mortality and it could generate adverse events and delay the administration of therapeutic measures that could improve outcomes^20–25^. However, in our country it is part of the initial management within hospitals.

Cholinergic and sedative/hypnotic toxidrome was the most frequent in our study. This result is related to the secondary effects of organophosphates and depressant medications on the central nervous system. However, many cases could not be investigated because it was not properly documented in the clinical records.

Our mortality was similar to the other countries in Asia and Africa, which reported a mortality of 4–8%^8,26^. However, in European countries (such as Spain), the mortality has been lower (0%)^11^. In 2021, the USA reported a mortality of 0.22%, and the principal causes of these deaths were acetaminophen (8.94%), miscellaneous hypnotics/sedatives/antipsychotics (8.08%), alcohols (7.73%), and pharmaceutical/illegal opioid preparations (7.59%)^9^. Kaya et al. reported a mortality of 1.1%^14^. Perhaps, the mortality is higher in our study due to the group of poisoning agents. In our group, the mortality in the pesticide poisoning group was 4.5%, lower than reported in some studies (8%)^26,27^. However, our mortality was higher compared to one study in the USA (0.07%)^28^. These studies did not identify pesticide poisoning, which explains the difference between our outcomes. Another reason could be associated with access to health services, the opportunity for care, and response capacity better than in our country.

### Context in Colombia

Previous studies conducted in different regions of the country reveal shared characteristics, although notable differences also emerge.

In 2010, a descriptive study investigated poisoning trends in Colombia using data from the Public Health Surveillance System^29^. A total of 23.844 cases were documented, with the urban population representing the predominant demographic group. Men constituted a significant proportion of poisonings, primarily involving pesticides and medications. Suicide attempts were the most frequent events, predominantly occurring through oral ingestion. These findings were similar to our research results.

In two descriptive studies, one performed by Vargas et al. utilizing data from the municipal health secretariat of a city in the center of the country during the period between 2009 and 2014^30^, and another study performed by Gutiérrez et al. using public health data of the municipalities in a western city during 2014^31^, described the epidemiological situation of poisoning in each of these in these respective regions. Despite variations in the number of cases examined in each study (5208 cases—440 cases), the characteristics were similar in both, characterized by a high prevalence in the urban population and predominantly affecting males. The distribution of cases displayed remarkable similarities concerning the type of substances involved and the nature of exposure, evident in both urban and rural populations. Nevertheless, Vargas found that psychoactive substances were the most frequently employed agents, in contrast to Gutiérrez, who identified chemical substances as the primary causative factors. In our study population, pesticides emerged as the predominant cause. This discrepancy might be attributed to the fact that the clinical management of organophosphate poisoning necessitates intensive care unit support, and our hospital stands as a referral center in southwestern Colombia. Additionally, the exclusion of the pediatric population in our study could be a contributing factor. Considering their unique characteristics, this demographic might have increased accessibility to medications or other chemical substances within their households.

From 2013 to 2016, Guerra-Rodríguez et al. conducted a study aiming to ascertain the correlation between intentionality and acute pesticide poisoning in patients admitted to a hospital in the western region of the country^32^. The study documented a total of 134 patients, primarily men residing in urban areas with lower educational attainment, revealing an association with suicidal intentions. Organophosphates were the most frequently employed substances, with half of the cases having a history of previous suicide attempts and one-third of the total cases diagnosed with a mental illness. These findings are concordant with our study, except for the exclusion of pediatric patients.

In 2018, a report on poisoning events aimed to characterize risk groups, alert situations, and outbreaks in our country^33^. The analysis included a total of 23,258 cases. Intoxications involving psychoactive substances and medications were particularly prevalent among individuals under 19 years of age. Males, especially those with lower educational levels in the urban population, constituted the majority of cases. The type of exposure was predominantly accidental and recreational. Mortality was reported in less than 1% of the population and was associated with pesticide poisoning. Compared to our study, there is a notable discrepancy in the primary cause with a lower mortality rate. The predominant exposure in our patient cohort was to pesticides, potentially explaining a higher proportion of deaths due to complications in this subgroup. However, it is essential to acknowledge the possibility of under-reporting, as the surveillance system lacks real-time reporting capabilities.

The population was mainly made up of women. The predominant educational level was secondary school. The oral route was the most used. For this year, mortality is still below 1% as in previous years. There is a difference in terms of gender and the most frequent group of substances in our study. The pandemic that began in 2020 generated changes in the distributions of the characteristics of the affected people.

In the 2020 poisoning report in Colombia^34^, a total of 33,029 poisoning cases were documented, with 15,697 records attributed to suicide attempts. More than half of the affected population comprised women, particularly those with a low educational level (secondary school). The oral route was the most common mode of exposure. The mortality rate remained below 1%, consistent with previous years. Gender characteristics and the primary group of substances in our study contrasted with the national findings for this period. The onset of the 2020 pandemic resulted in shifts in the demographic characteristics of affected individuals.

In the latest national poisoning report for week 13 of 2022^35^, a total of 44,071 poisoning cases were recorded, with 24,600 linked to suicide attempts. In contrast to our study, men reported the highest number of chemical substance poisonings, aligning with our findings. However, the female gender reported the highest proportion of poisoning cases related to suicide attempts, differing from our population due to the elevated proportion reported in men. The national report identified medications as the primary cause, followed by psychoactive substances and pesticides. In our findings, however, pesticides were identified as the primary cause. The potential impact of the pandemic on mental health may be associated with the rise in these substances as a cause of intoxication. Further studies are warranted on this matter.

Our results showed that the first cause of poisoning were medicines, followed by pesticides, chemical substances, and psychoactive substances. There is variability in the possible causes of intoxication in the different places, and it is related to the self-injurious intention and the availability or ease of accessing the substances. In our environment, pesticides and medicines, despite being regulated by government entities^36^, can be obtained over the counter, explaining the use of these agents. Given that the department of Valle del Cauca is one of the four departments in agricultural production according to the latest report of the National Agricultural Survey^37^, this could explain the frequent use of this group of toxic agents in our population.

The highest proportion of patients who attended our emergency room were transferred from first-level hospitals. According to the sustainability reports of our hospital, the percentage of patients affiliated with contributory or subsidized regimes was 75%, likewise with low and medium socioeconomic status. Our university hospital receives more than 60,000 patients from southwestern Colombia.

Our hospital is a reference hospital in southwestern Colombia due to its logistical, human resource, and technological capacity in medical care of serious diseases. It documented 91,000 patients attended the emergency room during 2022 (4% growth compared to the previous year)^38^.

One of the strengths of our study was a large sample of patients diagnosed with acute poisoning. We could obtain adequate quality data in the electronic medical records due to a straight review in the database.

This retrospective study was limited to a single hospital center. There were missing data related to the care times and medical management (decontamination management) due to first and second-level hospitals not describing this information.

However, we obtained relevant data for the region that can serve as a basis for other studies and take measures to generate public health policies.

## Conclusion

In our study, the main causes of acute poisoning were drugs and pesticides, with a predominant etiology of organophosphates and depressants of the central nervous system. There was a significant predominance of young male patients with suicidal intent, low mental disorders, elevated unemployment rate, and similar mortality reported in other studies. This study improves the knowledge about acute poisoning in southwestern Colombian to carry out multicenter analytics studies.

## Data Availability

The data that support the findings of this study are available from Fundación Valle del Lili but restrictions apply to the availability of these data, which were used under license for the current study, and so are not publicly available. Data are however available from the authors upon reasonable request and with permission from Fundación Valle del Lili. The email to request the database is centrodeinvestigacionesclinicas@fvl.org.co.
